# Conductive 3D nano-biohybrid systems based on densified carbon nanotube forests and living cells

**DOI:** 10.1557/s43578-023-01163-x

**Published:** 2023-10-25

**Authors:** Roya Bagheri, Alicia K. Ball, Masoud Kasraie, Aparna Chandra, Xinqian Chen, Ibrahim Miskioglu, Zhiying Shan, Parisa Pour Shahid Saeed Abadi

**Affiliations:** 1 Mechanical Engineering-Engineering Mechanics, Michigan Technological University, Houghton, MI 49931 USA; 2 Health Research Institute, Michigan Technological University, Houghton, MI 49931 USA; 3 Chemical Engineering, Michigan Technological University, Houghton, MI 49931 USA; 4 Materials Science and Engineering, Michigan Technological University, Houghton, MI 49931 USA; 5 Kinesiology and Integrative Physiology, Michigan Technological University, Houghton, MI 49931 USA; 6 Biomedical Engineering, Michigan Technological University, Houghton, MI 49931 USA

**Keywords:** Carbon nanotube forest, Conductive nano-biohybrid systems, Cardiomyocytes, Fibroblasts, Gelatin, Densification, Cell scaffold

## Abstract

Conductive biohybrid cell-material systems have applications in bioelectronics and biorobotics. To date, conductive scaffolds are limited to those with low electrical conductivity or 2D sheets. Here, 3D biohybrid conductive systems are developed using fibroblasts or cardiomyocytes integrated with carbon nanotube (CNT) forests that are densified due to interactions with a gelatin coating. CNT forest scaffolds with a height range of 120–240 µm and an average electrical conductivity of 0.6 S/cm are developed and shown to be cytocompatible as evidenced from greater than 89% viability measured by live-dead assay on both cells on day 1. The cells spread on top and along the height of the CNT forest scaffolds. Finally, the scaffolds have no adverse effects on the expression of genes related to cardiomyocyte maturation and functionality, or fibroblast migration, adhesion, and spreading. The results show that the scaffold could be used in applications ranging from organ-on-a-chip systems to muscle actuators.

**Graphical abstract:**

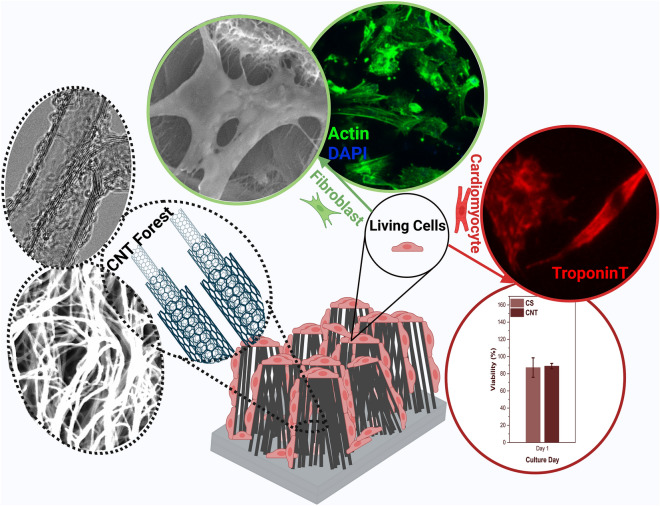

## Introduction

Biohybrid materials composed of conductive synthetic components and living cells have applications ranging from therapeutics to biorobotics and bioelectronics [1–10]. The conductive material provides the possibility of electrically controlling the functions of the cells or continuous read-outs and sensing. This capability is not provided by the commonly used hydrogel scaffolds with low conductivity. Such biohybrid systems are particularly advantageous for robotic systems, such as swimmers composed of beating cardiomyocytes integrated with control systems [11, 12]. Also, they are useful for organ-on-a-chip systems that are used for monitoring cell bioelectrical activities [13, 14].

Metal particles, conducting polymers, and carbon nanomaterials are three major categories of conductive materials that can be integrated with living cells on their own, when mixed with other non-conductive polymers, or in combination with a biomaterial coating [15–28]. However, all of the explored conductive scaffolds to date are materials within the lower end of conductivity, such as hydrogels mixed with small amounts of conductive substances or highly conductive materials that have a 2D sheet-like structure. The development of a conductive tissue scaffold with a 3D structure is needed to mimic the 3D native environment of the cells and simultaneously provide controllability through electrical signals.

Carbon nanotubes (CNTs) are a unique member of the family of carbon nanomaterials; they are one-dimensional cylindrical nanostructures with high electrical conductivity in the order of 10^4^–10^5^ S/cm [29]. Integrated with hydrogels such as gelatin methacrylate (GelMa), they have been used for the engineering of cardiomyocytes [30] and the fabrication of bioactuator swimmers [11]. Additionally, 2D sheets of horizontally-aligned CNTs have been used as a cardiac patch [31].

Previously investigated conductive scaffolds lack a 3D structure that could mimic the dimensionality of the native environment of cells. A possible alternative is a CNT forest. CNT forests are an excellent example of synthetic porous and fibrous materials; they comprise multi-walled CNTs grown on a substrate in a nominally vertical direction. Their height could range from tens of microns to cm scale. Although individual CNTs have a high Young’s modulus in the range of 300 GPa to over 1 TPa [32, 33], cumulatively as a CNT forest, they are compliant in the compressive mode with elastic modulus measured through indentation experiments to be in the range of 10–120 MPa [34–37]. Bending and buckling of CNTs are the dominant modes of deformation in compression. The mode and location of deformation depend on the growth parameters, coatings, and medium [38–43]. These ranges of stiffness make the material suitable as a cell scaffold without compromising the mechanical integrity and strength of the structure for integrated devices and systems. Furthermore, the large surface area of the CNT forest provides ample space for interaction with ions electrochemically, which is evidenced by the reported capacitance and impedance of CNT forests at 2.85 pF and 4700 W, respectively [44].

Here, the objective was to develop a 3D structured conductive scaffold for the growth and spreading of cells, especially cardiomyocytes. CNT forest was chosen due to the unique combination of morphology, conductivity, and mechanical properties. We used a thin layer of gelatin coating, which produced densified islands of CNTs due to surface tension forces. Additionally, the soft and biotic layer of gelatin enhanced the cytocompatibility of CNT forests. Moreover, the thickness was low to minimize an adverse effect on the electrical conductivity. We characterized the relevant morphological characteristics and properties of the scaffolds. Then, we used NIH-3T3 mouse fibroblasts to mainly show the cytocompatibility and cardiomyocytes from neonatal rat hearts to demonstrate the efficacy of the material as a cardiac cell scaffold. The behaviors of the cells on the CNT forest scaffolds were studied through toxicity studies, immunofluorescent imaging, electron microscopy, and quantification of gene expression. The novelty of the work lies in the use of the 3D structure of CNT forests as the main part of the scaffold and the development of a conductive, porous, and 3D cardiac scaffold with high cytocompatibility.

## Results

### Fabrication and physical properties of the CNT forest scaffold

**Figure 1:**
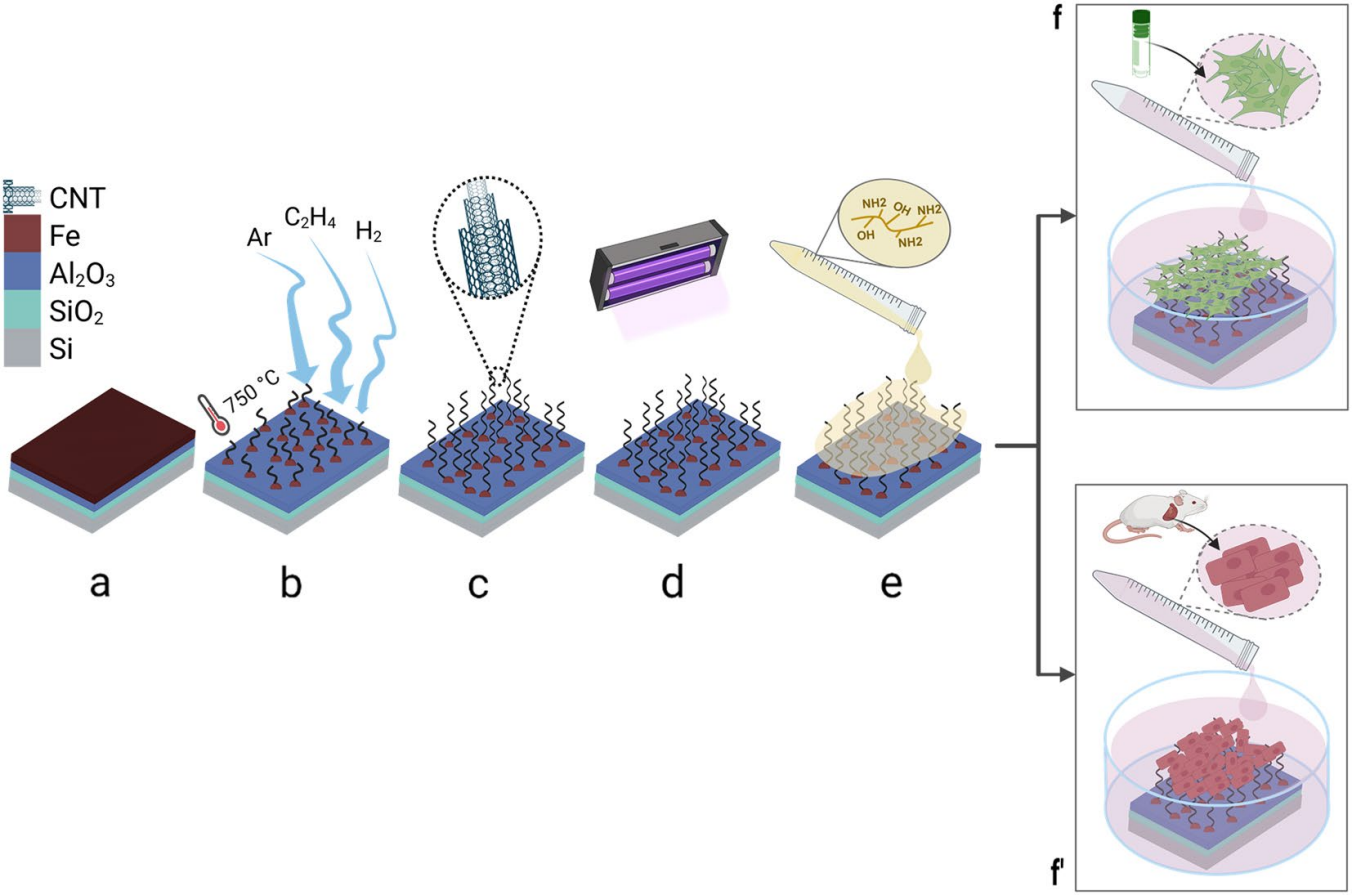
Schematic illustration of the process from CNT forest growth to cell seeding: (a) Silicon wafer coated with SiO_2_, Al_2_O_3_, and Fe as catalyst, (b) Exposure to ethylene, argon, and hydrogen gases for the growth of CNT forests, (c) CNT forest after the termination of growth, (d) Sterilization of CNT forests using 70% ethanol (not shown) and UV exposure, (e) Coating of CNT forests with gelatin, and (f, f^’^) Cell seeding of fibroblasts derived from a NIH 3T3 mouse cell line (f) or primary cardiomyocytes extracted from neonatal rat heart (f^’^).

The CNT forests were grown using chemical vapor deposition (CVD) on 1 cm × 1 cm Si wafer pieces coated with SiO_2_, Al_2_O_3_ (alumina), and Fe [Fig. 1(a)]. The Fe film breaks into nano-sized islands in the process of heating the substrates to 750 °C, whereas the alumina support layer assists in the narrow distribution of the size of the catalyst islands leading to the formation of high-density forests and vertical alignment through proximity effects [45]. CNT growth was carried out with ethylene, argon, and hydrogen gases [Fig. 1(b)]. Ethylene is the source of carbon atoms that make up the nanostructure of CNTs, and the other two gases function as carrier gases in the growth chamber. The CNT forests grew in a nominally vertical direction [Fig. 1(c)] and showed wavy and entangled CNTs. After growth, the CNT forests were sterilized using 70 vol% ethanol and UV light exposure [Fig. 1(d)]. In the following steps, the sterilized CNT forests were coated with a thin layer of gelatin [Fig. 1(e)] and were incubated in the cell culture medium at 37 °C and 5% CO_2_ for at least 2 h before cell seeding [Fig. 1(f)].

**Figure 2:**
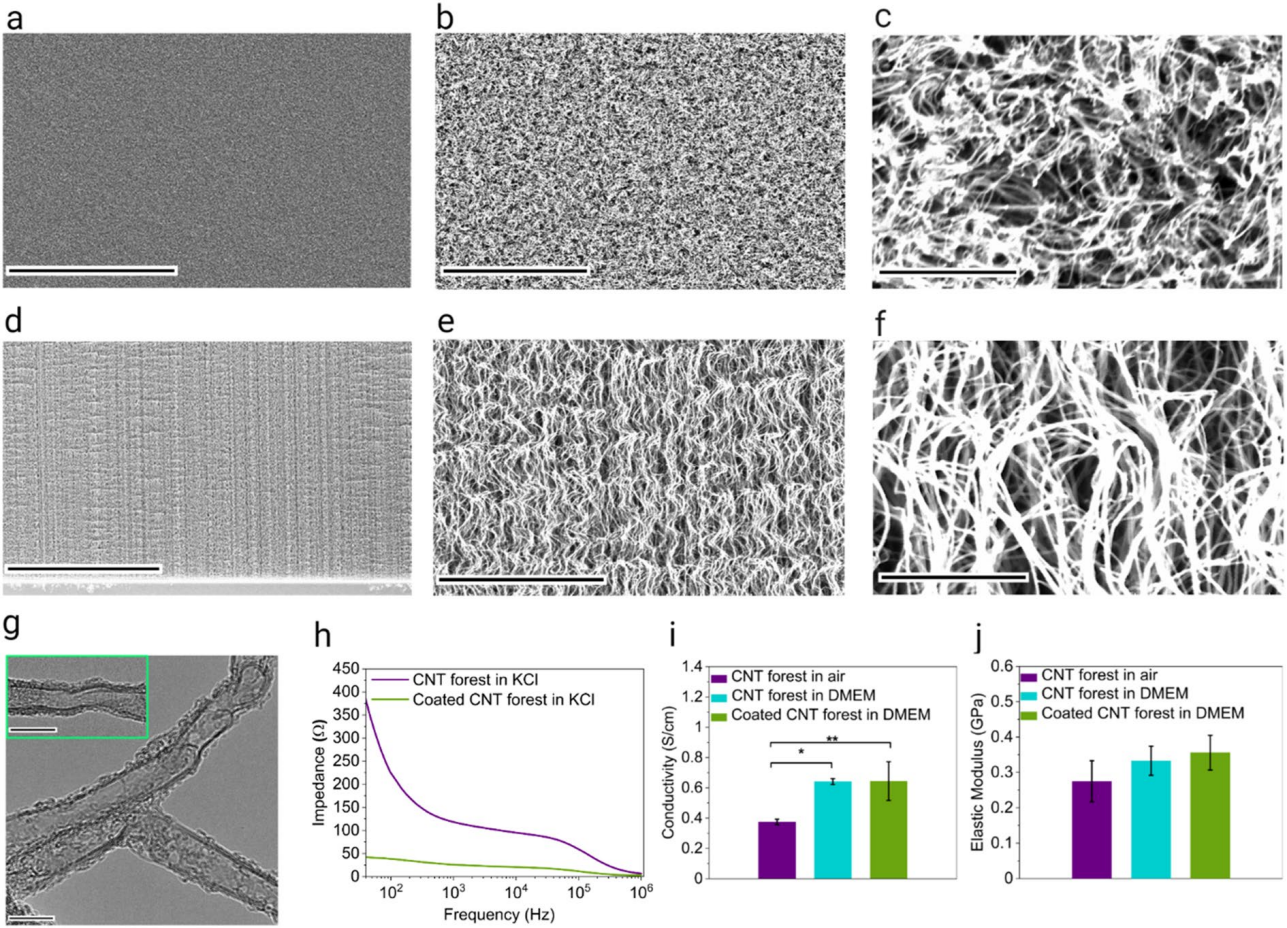
Physical characterization of CNT forests: (a–c) SEM images of the top view of a dry CNT forest at different magnifications, scale bars, 100 µm, 10 µm, and 100 nm, respectively; (d–f) SEM images of the side view of a dry CNT forest at different magnifications, scale bars, 30 µm, 4 µm, and 500 nm, respectively; (g) TEM images of CNTs from a CNT forest, scale bars, 10 nm; (h) Impedance vs. frequency; (i) Electrical conductivity, (*p < 0.05, **p < 0.001); (j) Elastic modulus.

We used scanning electron microscopy (SEM), transmission electron microscopy (TEM), four-point probe, electrochemical impedance spectroscopy, and nanoindentation to characterize the CNT forests. The CNTs were wavy and entangled [Fig. 2(a–f)], with a height in the range of 120–240 µm as measured by SEM. A diameter of 13.7 ± 0.5 nm was measured by using TEM images, and a bamboo-like nanostructure was observed [Fig. 2(g)]. Impedance decreased with the gelatin coating due to the enhancement of connections between the CNTs through the gelatin film, leading to improved transfer of charge carriers such as electrons and ions [Fig. 2(h)]. Electrical conductivity increased by soaking the CNTs in the culture medium [Fig. 2(i)]. We speculate that the ion transfer through the liquid medium plays a role in the higher measured current, as the cell culture medium is an ionic liquid composed of sodium, potassium, calcium, magnesium, etc. Nanoindentation using a Berkovich tip (*i.e.,* a three-sided pyramid) showed no statistically significant change of elastic modulus by immersing the CNT forests in the culture medium or by coating them [Fig. 2(j)]. The mode of deformation is the bending of the tip of the CNTs, which is expected to be similar to the deformations caused by the attachment of cells to the CNT forests and by the contraction of cardiomyocytes. The elastic modulus was in the range of 0.2–0.4 GPa, which is three orders of magnitude smaller than conductive 2D sheets, such as graphene [46, 47].

### Cardiomyocyte and 3T3 fibroblasts’ viability and organization on CNT forest scaffolds

**Figure 3:**
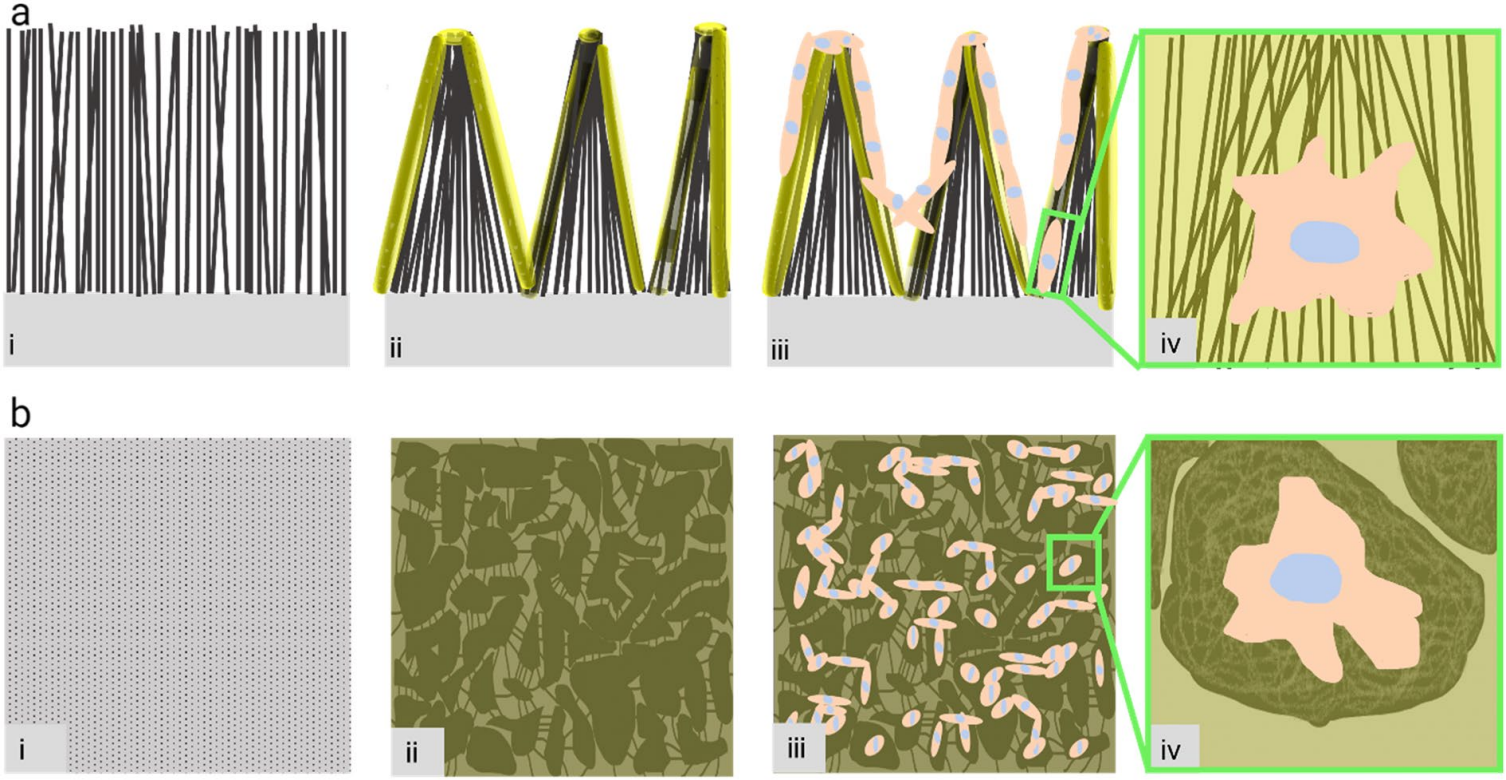
Schematic illustration of the formation of 3D cell networks on CNT forest scaffolds: (a) side view; (b) top view; (i) CNT forest in air; (ii) CNT forest coated with gelatin in the growth medium; (iii, iv) CNT forest scaffold seeded with cells (either fibroblasts or cardiomyocytes) at low (iii) and high (iv) magnifications.

Due to the gelatin coating on top of the CNT forest, CNTs are bent and form densified islands, as shown schematically in Fig. 3. The densification is similar to the case of densification due to the evaporation of liquid from CNT forests [48–50] and surface tension in the liquid. For the case of gelatin coating of CNT forests, we believe that a similar equilibrium mechanism causes densification and the resulting morphology. A likely scenario is that interactions between the gelatin and CNTs initiated tears in the gelatin layer. Physical wrapping and hydrophobic interactions [51] are expected to be dominant at the interface of CNTs and gelatin because no crosslinking agent or surfactant was used. Due to the nominally vertical direction of the CNTs, the horizontal gelatin film on top experienced high levels of stress at contact points with the tips of CNTs, which led to formation of defects such as holes and tears in one or more locations. The tears propagated to the entire surface of the scaffold. The shrinkage of separated pieces of gelatin then applied bending loads on the CNT forests and patterned them. The produced patterned surfaces changed the appearance of the CNT forest from a monolithic porous film to a 3D structure composed of micron-scale densified islands (Fig. 3). According to several investigations of mechanical properties and visualization of deformation of CNT forests, the compressive modulus is in the range of 10–120 MPa and CNTs easily bend due to their high aspect ratio [34–37]. Low stiffness and being prone to bending facilitate the formation of densified islands of CNTs within the scaffolds due to interactions with the gelatin coating.

**Figure 4:**
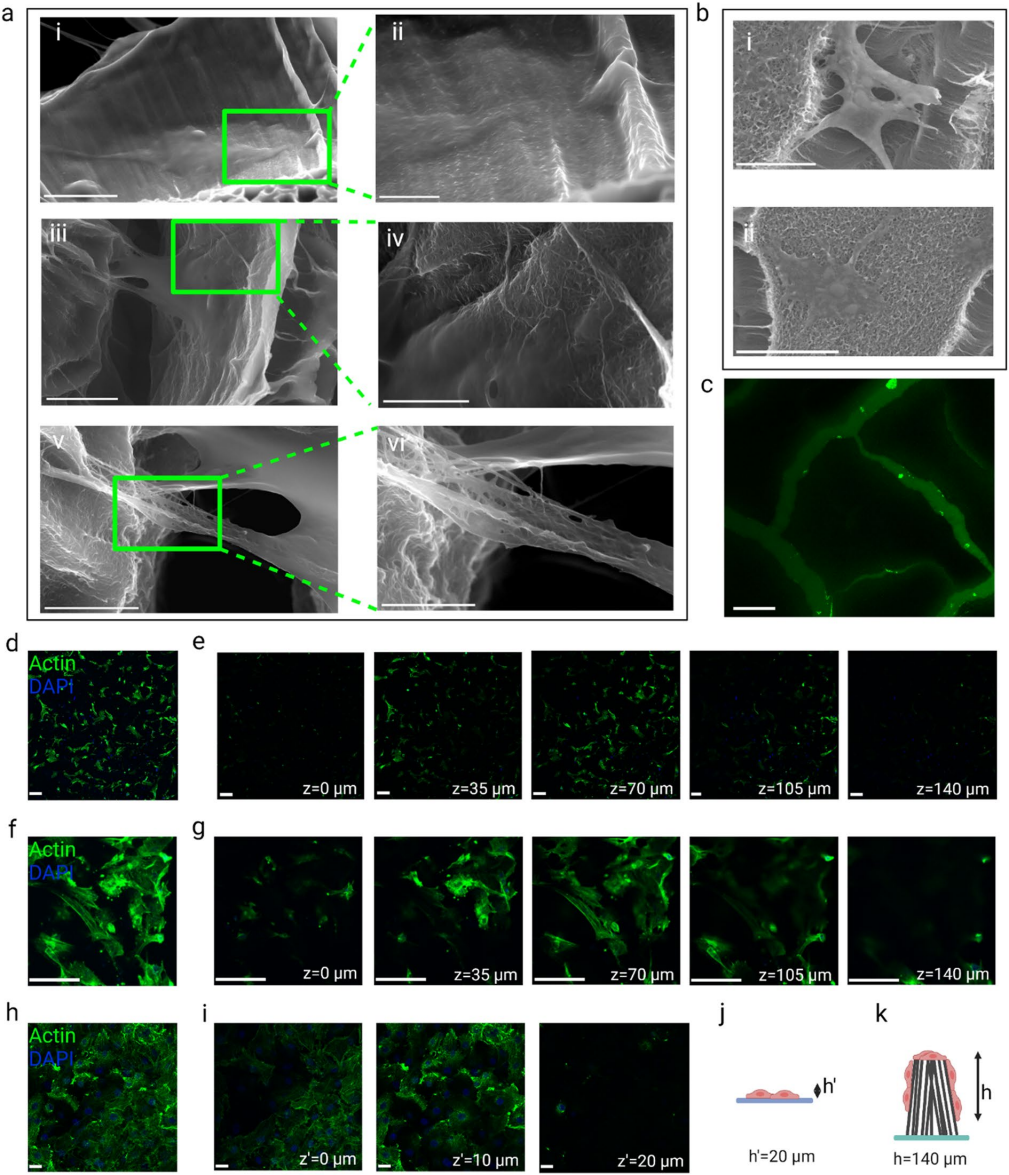
Interactions of fibroblasts with the 3D structure of the CNT forest scaffolds; (a) ESEM images of a CNT forest scaffold seeded with fibroblasts on day 3, (i, ii) a cell attached to the CNT forest wall, scale bars, 20 and 5 µm respectively, (iii, iv) a cell connecting two CNT forest walls, scale bars, 10 and 3 µm, (v, vi) a cell connection to CNTs, scale bars, 4 and 2 µm respectively; (b) (i) An SEM image of a fibroblast (day 15) attached to the walls of densified regions of a CNT forest scaffold bridging the open space, scale bar, 20 µm, (ii) An SEM image of the top surface of a CNT forest scaffold and a fibroblast (day 15) spread on it, scale bar, 40 µm; (c) Calcein AM staining of fibroblasts (day 3) on CNT forest scaffolds with the background staining showing the densified forests and the location of cells on the side walls of the densified regions, scale bar, 100 µm; (d–g) Immunofluorescence micrographs of fibroblasts on CNT forest scaffolds (day 7) labeled with phalloidin for F-actin (green) and DAPI for nuclei (blue) showing z-stack confocal images at low (d) and high (f) magnifications and their deconstruction into 2D images (e and g corresponding to d and f, respectively) covering a z range of 140 µm showing the spreading of the cells along the height of CNT forests, scale bar, 100 µm, (i) Immunofluorescence micrographs of fibroblasts on coverslip (CS) as control (day 7) labeled with phalloidin for F-actin (green) and DAPI for nuclei (blue) showing a z-stack confocal image (h) and it’s deconstruction into 2D images (i) covering a z range of 20 µm showing the 2D nature of scaffold, scale bar, 100 µm; (j, k) Schematic illustration of the 2D and 3D morphology of fibroblasts on coverslip and CNT forest scaffold, respectively.

**Figure 5:**
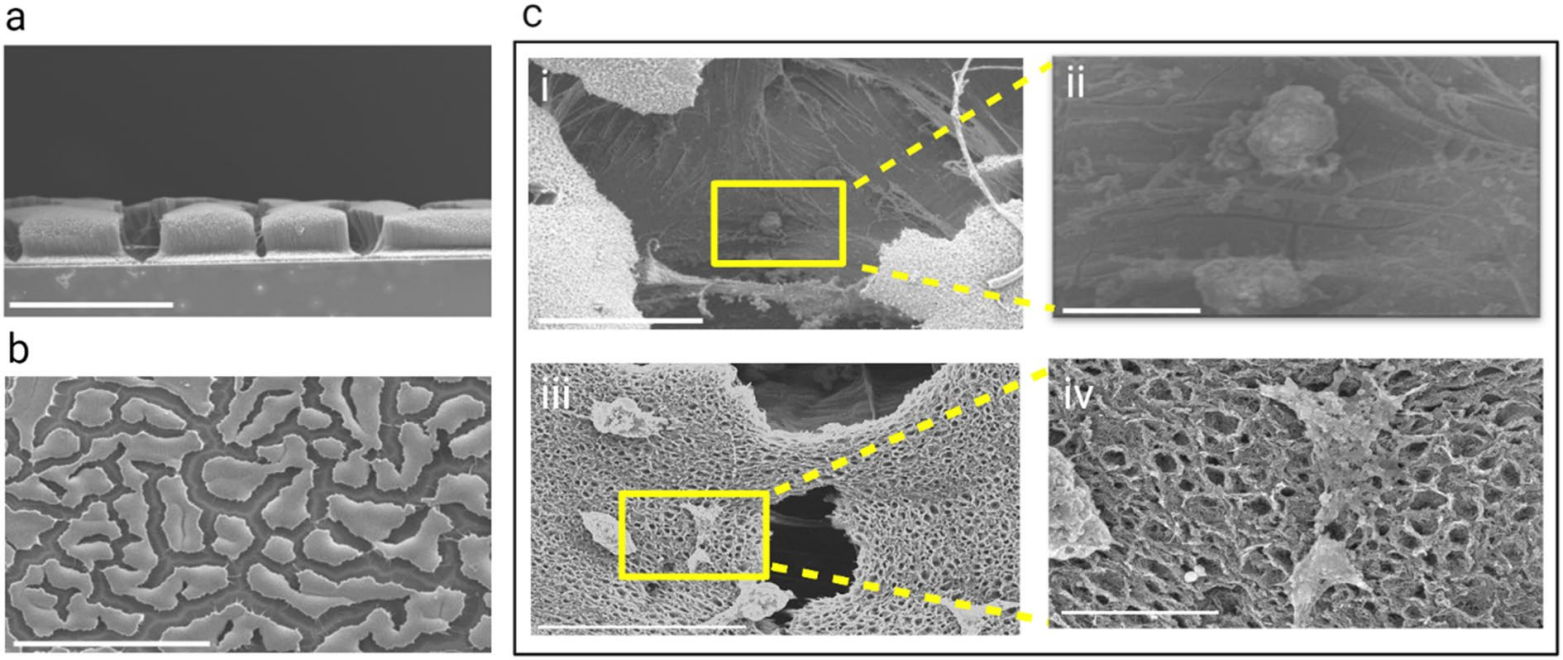
Interactions of cardiomyocytes with the 3D structure of the CNT forest scaffolds: (a, b) SEM images of the side and top views, respectively, of a densified CNT forest scaffold seeded with cardiomyocytes on day 14 and 15, scale bar, 100 and 500 µm respectfully; (c) Higher magnification SEM images, (i, ii) higher magnification images of a cell in the space between densified CNT forest regions, scale bars, 50 and 10 µm respectively, (iii, iv) higher magnification SEM images of cells spread on top of the scaffold, scale bars, 40 and 5 µm respectively.

After coating CNT forests with gelatin, cardiomyocytes and fibroblasts were seeded on them. The rationale for the examination of two cell types was to improve our understanding of the effect of the scaffold on cells and acquire knowledge not limited by the type of cell. Controls included common tissue culture surfaces, glass coverslip (CS) for all tests, and tissue culture polystyrene (PS) in addition to CS for a toxicity test. Although the PS (standard coating of tissue culture well plates) was used as a control for the PrestoBlue toxicity testing, it was not a convenient option for any characterization by imaging. The reason is the difficulty of imaging the bottom of the tissue culture well plates using an upright fluorescent microscope. Note that an inverted microscope could not be used because of the CNT forest specimens being opaque and possible adverse effects of imaging living cells at an upside down state. The controls went through the same sterilization and coating procedure as the CNT forest scaffolds. Imaging scaffold-cell constructs via SEM and immunofluorescence microscopy revealed morphology. Cells adhered to the top and side walls of the densified CNT forest islands and formed a 3D network as schematically depicted in Fig. 3. SEM images further proved the spreading of cells on top and in between the densified regions of CNT forests [Fig. 4(a, b)]. Environmental SEM (ESEM), which uses a higher pressure and humidity than a regular SEM, shows the morphology in an environment more similar to the native environment [Fig. 4(a)]. Immunofluorescence imaging of the scaffolds stained with Calcein is evidence of the creation of these islands [Fig. 4(c)] in the wet state, showing that the densification phenomenon is not an artifact of the dehydration and drying steps in the SEM sample preparation. The presence of cells along the height of CNT forests was confirmed and the depth was quantified by acquiring z-stack confocal images. In a typical CNT forest scaffold, over 100 µm was covered with cells, whereas the height of cells on coverslips did not exceed 20 µm [Fig. 4(d–k)]. For the case of cardiomyocytes, the densification of CNT forests as well as the formation of 3D networks of cells on CNT forests were observed in SEM micrographs (Fig. 5). Densified CNT forest islands were observed in the dry state through SEM [Fig. 5(a, b)]. Cardiomyocytes were spread on top and in between the CNT forest islands [Fig. 5(c)].

**Figure 6:**
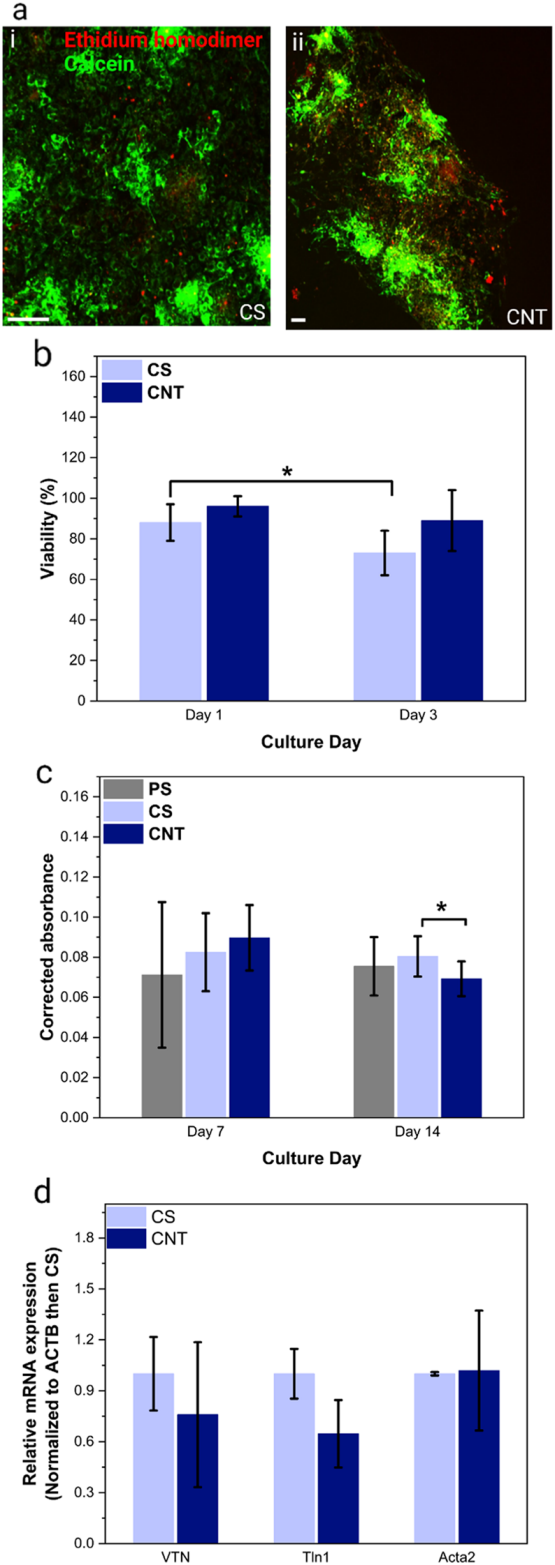
Organization, viability, and gene expression of fibroblasts on CNT forest scaffolds: (a) Live-Dead immunofluorescence micrographs of fibroblasts (day 3) on the CS as control (i) and CNT scaffold (ii), scale bars, 200 µm; (b) Viability of fibroblasts on CNT forest scaffolds and CS as control calculated by the ratio of the number of live cells to total cells in the Live-Dead stained micrographs (*p < 0.01); (c) Absorbance data obtained through the Presto-Blue assay (*p < 0.05) representing the viability of cells; (d) Expression of VTN, TLN1, and ACTA2 genes representing adhesion, spreading, and migration of fibroblasts on day 3 on CNT forest scaffolds and CS as control (normalized to ACTB then CS) showing no statistically significant difference.

The cytocompatibility of CNT forest scaffolds with fibroblasts and cardiomyocytes was assessed through the live/dead two-color fluorescence cell viability assay and by using the PrestoBlue reagent, which functions by the measurement of the reducing power of living cells. In the former, the results were compared with CS as control, whereas in the latter, CS and PS were used as controls. The viability assessment of fibroblasts using the two-color fluorescence assay [Fig. 6(a)] indicated 96% viability on day 1 and 89% on day 3 [Fig. 6(b)]. The viability of fibroblasts on the CNT forest scaffolds and CS did not show any significant difference with CS as a control on days 1 and 3, which indicates that the CNT forest scaffold does not have any toxicity. The PrestoBlue assay [Fig. 6(c)] showed no toxicity for the scaffolds with fibroblasts on day 7 compared with controls, which can be related to the chemically inert nature of CNTs, coating with gelatin, and anchoring of CNTs to the substrate [30, 52, 53]. However, 12% lower viability was measured on day 14 for CNT forests compared with CS, showing a low level of toxicity, possibly due to the inhibition of proliferation and programmed death of some fibroblasts on CNT forests. To compare the extracellular modulation and cytoskeleton maturation of fibroblasts on CNT forest scaffolds and the controls, we performed real-time polymerase chain reaction (RT-PCR) to quantify the expression of the following genes: VTN, the gene encoding a glycoprotein linked to either cell adhesion or cell migration; TLN1, a gene encoding a cytoskeletal protein related to F-actin and to the cell migration and spreading; and ACTA2, a gene encoding an actin protein. The data on gene expression showed no significant difference between the expression of the examined genes in the fibroblasts on the CNT forest scaffolds and CS controls and showed no adverse effects of CNT forest scaffolds on the cytoskeleton, adhesion, spreading, or migration of the fibroblasts.

**Figure 7:**
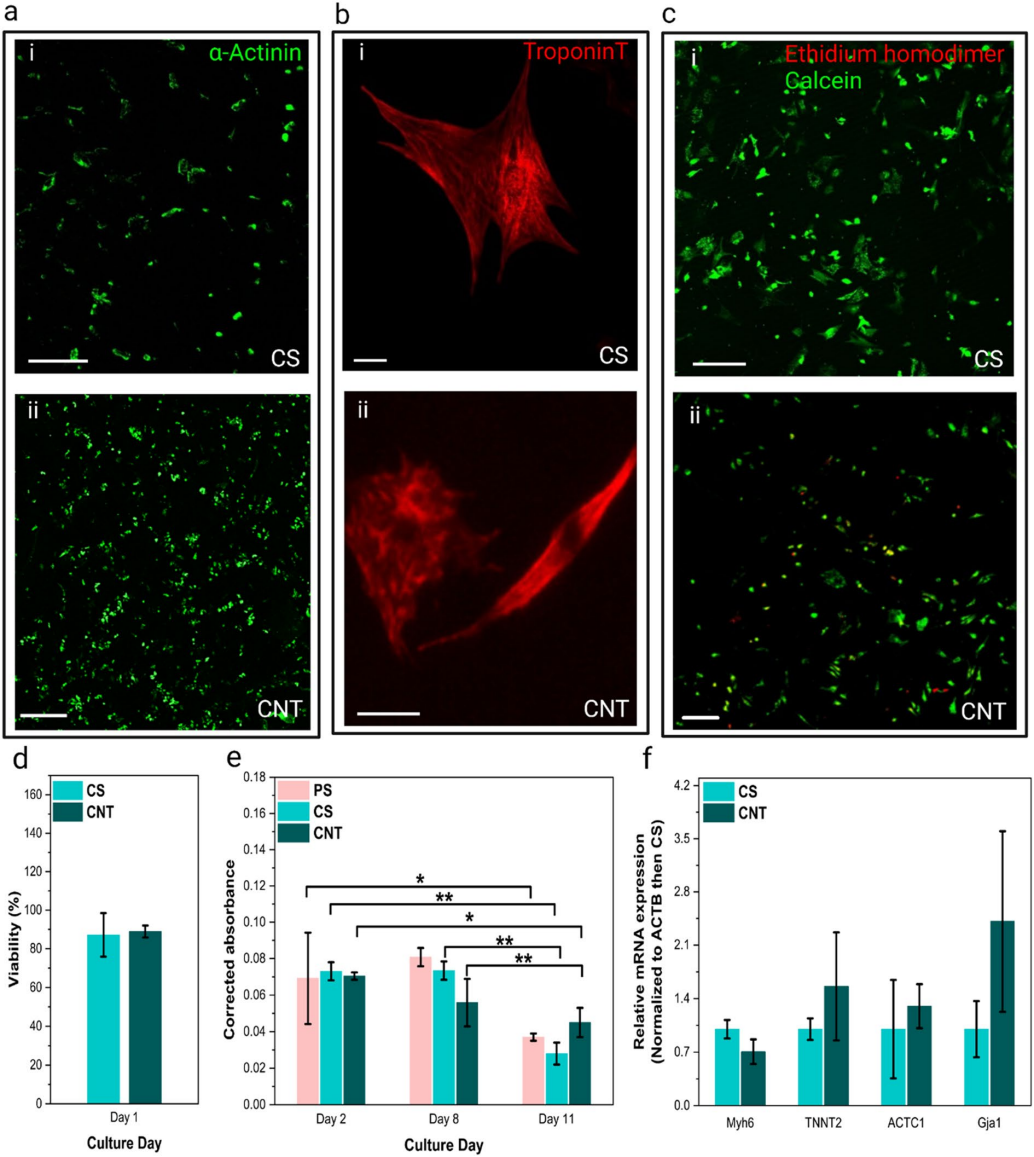
Organization, viability, and gene expression of cardiomyocytes on CNT forest scaffolds: (a) Immunofluorescence micrographs of cardiomyocytes labeled with monoclonal anti-α-actinin (sarcomeric) on a CS (i) and a CNT forest scaffold (ii); day 14; scale bars, 200 µm; (b) Immunofluorescence micrographs of cardiomyocytes labeled with anti-cardiac troponin on a CS (i) and a CNT forest scaffold (ii); day 19; scale bars, 20 µm; (c) Live-Dead immunofluorescence micrographs of cardiomyocytes (day 5) on a a CS (i) and a CNT forest (ii); scale bars, 200 µm; (d) Viability of cardiomyocyte on CNT forest scaffolds and CS as control calculated by the ratio of the number of live cells to total cells in the Live-Dead stained micrographs; (e) Absorbance data obtained through the Presto-Blue assay (PS & CS as control), (*p < 0.05, **p < 0.001) representing the viability of cells; (f) Expression of MYH6, TNNT2, ACTC1 and GJA1 genes representing maturation and functionality of cardiomyocytes at day 8 on CNT forest scaffolds and CS as control (normalized to ACTB then CS).

Confocal fluorescence imaging of cardiomyocytes labeled with cardiac markers, α-Actinin, an indicator of actin-binding proteins, and troponin T, an indicator of calcium-binding proteins, was used to qualitatively compare the expression of cardiac markers for cells on CNT forest scaffolds and CS controls and to assess the organization of cells [Fig. 7(a, b)]. For cardiomyocytes, the two-color fluorescence viability assay [Fig. 7(c)] indicated 89% viability for the scaffold on day 1, which was in the range with the control [Fig. 7(d)], showing that the scaffold does not have any toxicity. The PrestoBlue assay showed no significant difference between the viability of cells on the scaffolds and the controls on any of the 3 time points [Fig. 7(e)]. Similar to the case of cytocompatibility of CNT forest scaffolds with fibroblasts, the cytocompatibility here can be related to the chemically inert nature, coating, and anchoring of CNTs to the substrate [30, 52, 53]. On day 11, as expected, viability decreased compared with the earlier time points, which is consistent between the CNT forest scaffolds and the controls. The difference between the cytocompatibility at the later time point between the two cell types is likely due to the higher rate of proliferation of fibroblasts compared with cardiomyocytes; the CNT forest might cause a decrease in the rate of proliferation of fibroblasts compared with the control scaffolds, which is not a major issue for cardiomyocytes. Furthermore, the expression of the following genes was evaluated quantitatively through RT-PCR on day 8: MYH6, the gene instructing the production of alpha heavy chain subunit of cardiac myosin, which is the molecular motor powering contractions of cardiomyocytes; TNNT2, the gene instructing the production of the troponin complex in the sarcomere structure responsible for contraction of cardiomyocytes; ACTC1, the gene encoding the protein alpha actin, which is a major part of the cardiac contractile apparatus; GJA1, the gene instructing the production of protein connexin 43 for cell-to-cell communication through gap junctions. The RT-PCR data showed no significant difference between the expression of cardiac markers for cells on the CNT forest scaffolds compared with controls [Fig. 7(f)].

## Conclusions

We developed a conductive nano-biohybrid system comprising living cells integrated with 3D structures of densified CNT forests coated with a thin layer of gelatin. NIH 3T3 fibroblasts and primary rat cardiomyocytes were used. Densification of the CNT forest, which occurred due to mechanical stresses in the gelatin layer adhered to the surface of the CNT forest, resulted in islands of bent CNTs. Cells—both fibroblasts and cardiomyocytes—were found attached to the surface and side walls of the CNT forest islands, thus forming a 3D network of living cells, as revealed by fluorescence confocal micrographs and SEM (environmental and high vacuum). Up to day 11, the scaffold showed no significant toxicity on either cell. On day 14, a 12% lower viability of fibroblasts was measured on CNT forest scaffolds compared with the controls, showing a mild level of toxicity, possibly due to slightly lower proliferation and higher programmed death of fibroblasts on the CNT forests. Importantly, the CNT forest scaffolds did not change the genotype of the cells in several tested genes, including those related to the contractile apparatus and the cell-to-cell communication in the case of cardiomyocytes and adhesion, migration, and spreading in the case of fibroblasts. Thus, in summary, we fabricated a cytocompatible 3D conductive biohybrid system with no observed adverse effects on the functions of the cells. The controllability of the cells through electrical signaling and the 3D morphology make the system applicable to biorobotics and bioelectronics.

## Experimental section

### Materials

Gelatin methacryloyl was purchased from Sigma-Aldrich, USA. NIH 3T3 fibroblasts, Dulbecco’s modified Eagle’s medium (DMEM), phosphate buffered saline (PBS), and penicillin–streptomycin (Pen/Strep) were purchased from ATCC, USA. Heat-inactivated fetal bovine serum (FBS) and a Pierce™ primary cardiomyocyte isolation kit were purchased from Thermofisher, USA. Silicon (Si) wafers with (100) orientation were purchased from University Wafer, Inc., USA. They had a 1-µm thermally grown SiO_2_ coating, resulting in a total thickness of 500 µm. PBS-Pen/Strep was prepared by mixing 5 ml of Pen/Strep with 500 ml of PBS (~ 1 vol%).

### CNT forest growth

CNT forests were grown on Si wafers (1 cm × 1 cm) with a 1-µm layer of SiO_2_ coating by chemical vapor deposition (CVD) technique under atmospheric pressure in FirstNano^R^ EasyTube^R^ 101 (CVD equipment corporation, USA). An Al_2_O_3_ layer as a buffer and a Fe layer as a catalyst were deposited on Si wafers prior to CVD. To obtain the Al_2_O_3_ layer, first, 10 nm of Al was deposited by an E-beam evaporator (Frederick EB12, Frederick company, USA) with a deposition rate of 1 Å/s. Then, the substrate was annealed at 500 °C for 30 min to form Al_2_O_3_ films. Last, Fe (2 nm) was deposited by the same E-beam evaporator at a deposition rate of 1 Å/s. CNT growth was carried out at 750 °C with 230.5 sccm ethylene, 265.5 sccm argon, and 307.3 sccm hydrogen for 12 min.

### Sterilization and coating

The sterilization of CNT forests started with soaking the specimens in 70 vol% ethanol, followed by overnight ultraviolet (UV) light exposure with 40 µW/cm^2^ intensity at a distance of 724 mm. Then, the specimens were washed with PBS-Pen/Strep three times for 30 min each. The gelatin solution (1 wt%) was prepared by dissolving gelatin in PBS-Pen/Strep solution at 50 °C for 60 min. The gelatin solution was filtered using a 0.22 µm filter (Thermofisher, US). CNT forests were placed in the gelatin solution and incubated at 37 °C and 5% CO_2_ for 2 h. The gelatin solution was then removed, and the CNT forests were washed with PBS-PenStrep twice for 5 min each. The scaffolds were then placed in a cell culture medium relevant to the specific cell used (Dulbecco's Modified Eagle Medium (DMEM) (ATCC, US) for 3T3 and DMEM for primary cell isolation (with high glucose for cell isolation) (Thermofisher, US) for CM) and incubated at 37 °C and 5% CO_2_ for at least 1 h before cell seeding.

### Cell culturing

All animal protocols were approved by the Michigan Technological University Institutional Animal Care and Use Committee. 12-week-old male and female Sprague Dawley (SD) rats were purchased from Charles River Laboratories (Wilmington, MA, USA) and used in the breeding colony to generate pups. All rats were housed and kept on a 12:12 h light–dark cycle in a climate-controlled room. Chow and water were provided ad libitum. The 1–3-day-old pups were euthanized by an overdose of isoflurane, and the hearts were separated. A cardiomyocyte isolation kit was used for the isolation of cardiomyocytes from the hearts, and all of the manufacturer’s protocols were followed. Briefly, each heart was minced into 1–3 mm^3^ pieces and washed with 500 μl Hanks' balanced salt solution (HBSS). A cardiomyocyte isolation enzyme with papain (0.2 ml) and one with thermolysin (10 μL) were added to the heart pieces, which were then incubated at 37 °C for 30 min. After removing the enzymatic solution, the heart pieces were washed with 500 μL of HBSS. After adding 0.5 ml complete DMEM (DMEM with 10 vol% FBS and 1 vol% Pen/Strep), the heart pieces were dissociated into a single-cell suspension by pipetting ~ 30 times. Then, 1 ml of the complete DMEM was added to the cell suspension, and the cardiomyocytes were seeded on the scaffolds with a density of 3 × 10^5^ cells/cm^2^. The samples were cultured in a pre-warmed complete Primary Cell Isolation DMEM containing 10 vol% heat inactivated FBS, 1 vol% Pen/Strep, and 0.1 vol% cardiomyocyte growth supplement in DMEM for up to 14 days. The purpose of adding the cardiomyocyte growth supplement was to remove the fibroblasts and increase the purity of cardiomyocytes. The DMEM with cardiomyocyte growth supplement was changed every 3 days.

NIH 3T3 fibroblasts (ATCC Inc., USA) at passage 2 were used. Fibroblasts were seeded on the CNT forests and the controls with the density of 1 × 10^5^ cells/cm^2^. Complete DMEM was used as the cell culture medium and was changed every 3 days after cell seeding.

### SEM

The morphology and microstructure of the scaffolds were assessed in SEM. First, dry CNT forests were imaged in their original condition without any changes or coatings. Then, to image the cell-laden scaffolds, they were first washed three times with PBS for 10 min each, followed by fixation with 5 wt% aqueous glutaraldehyde for 2 h. Then, they were dehydrated with a series of ethanol solutions: 35, 50, and 75 vol% for 30 min each, 95 vol% for 60 min, and Absolute ethanol for 120 min. Following dehydration, specimens were placed in a desiccator under vacuum and allowed to dry overnight. To increase electrical conductivity, the samples were coated with a ~ 5-nm layer of Pt/Pd in a Cressington 208HR high-resolution sputter coater, in which the thickness of the coating was measured with an MTM-20 film thickness controller. The samples were imaged using a Hitachi S-4700 cold field-emission high-resolution SEM. SEM micrographs were taken at a working distance of 12 mm by using a beam voltage and current of 10 kV and 10 µA, respectively.

### ESEM

The attachment and morphology of 3T3 fibroblasts on the CNT forest scaffolds were investigated using ESEM (Quattro ESEM, Thermofisher, USA). The cells were fixed and prepared with the same protocol for the SEM fixation sample and were stored in PBS. The samples were under 700–750 Pa pressure, 79–87% humidity, a temperature of 5 °C, and an accelerating voltage of 15 kV. Images were collected with Thermo Scientific map software.

### TEM

TEM imaging of CNTs was performed using an FEI Titan Themis scanning TEM (STEM) operating at 80 kV. The images were acquired in an energy-filtered TEM mode at the zero loss energy. To prepare CNTs, we separated them from the Si substrates and dispersed them in ethanol (90% v/v in water) by bath sonication for 10 min (Branson 1800, Branson Ultrasonics, USA). Then, a drop was placed on a Cu TEM grid coated with a lacey carbon film and allowed to dry before imaging.

### Electrical characterization

The four point probe setup used four 1-mm spaced tungsten metal tips and a Keithley 4200A-SCS parameter analyzer. The voltage drop was measured across the two inner probes when passing a linearly sweeping current through the two outer probes. The slope of the *I*–*V* plot was used to determine the sample resistance and then electrical conductivity. A minimum of three measurements were done per sample, and 3 samples per group were tested (n = 3).

### Impedance

Electrochemical impedance measurements were performed with an Electrochemical Analyzer 600E (CH Instruments Inc., USA) using a conventional three-electrode cell. An Ag/AgCl wire (saturated in KCl) (CH Instruments Inc., USA) was used as a reference electrode, and a Pt wire (CH Instruments Inc., USA) was used as a counter electrode. The scaffolds as working electrodes were held inside the electrolyte using an alligator clip outside the liquid surface and connected to the wiring. Impedance measurements were performed in a 1 M KCl solution with an AC bias sweeping in a frequency range of 40 Hz to 1 MHz, with an amplitude of 5 mV (n = 3).

### Nanoindentation

Nanoindentation testing was performed in an Agilent NanoIndenter XP system using a Berkovich tip. Testing was performed on at least 3 points on each sample and 3 samples of each group (n = 3). Indentation was performed up to the depth of 500 nm with the strain rate of 0.05 S^−1^. For the case of samples immersed in the culture medium, a metal puck capable of holding a pool of the liquid was used and the samples stayed under the liquid surface for the entire duration of testing. All samples were attached to the metal holders using epoxy glue. Elastic modulus was calculated using the initial part of the unloading portion of the load–displacement curve by the Oliver-Pharr method [54]. The area of the Berkovich tip was determined using calibration specimens according to standard protocols.

### Cell viability

The viability of both 3T3 cells and cardiomyocytes was assessed on the scaffolds by live-dead and PrestoBlue assays. Calcein AM and Ethidium homodimer reagents (Thermofisher, US) were used, respectively, for labeling live and dead cells based on the manufacturer's instructions. The live-dead images of the cells were taken on day 1 for cardiomyocytes and on days 1 and 3 for 3T3 cells. The cell viability was calculated by dividing the number of live cells by the total number of cells counted in the images. Three samples for each configuration were imaged (n = 3), and at least three locations were imaged on each sample. In the second method, the PrestoBlue reagent (Thermofisher, USA) was added to each well, and the plates were incubated at 37 °C and 5% CO_2_ for 15 min. Following incubation and the removal of the PrestoBlue solution from samples, 100 µl of the solution was transferred to a new well in a 96-well Plate. The PrestoBlue reagent (blue color and non-fluorescent) was reduced by metabolically active cells (live cells) from resazurin (blue color) to resorufin (red color and highly fluorescent). Fluorescence was measured at the excitation/emission wavelengths set at 530/590 nm and 560/590 nm in a microplate reader (Biotek Synergy/HTX Multi-Mode, Biotek Instruments, Vermont, USA).

### Immunofluorescence microscopy

Fluorescence microscopy was performed on cardiomyocytes labeled with cardiac markers. First, the samples were washed three times for 5 min with PBS and then fixed with 4 vol% paraformaldehyde (PFA) solution (Electron microscopy, US) followed by three times wash with PBS for 5 min each. The samples were permeabilized with 0.1 vol% Triton X-100 solution in PBS for 15 min at room temperature. Then, donkey serum blocking solution (8 vol% in PBS) was added to the samples for 1 h at room temperature. The cells were labeled in a blocking buffer overnight at 4 °C, with two primary antibodies, α-actinin (Mouse, 1 mg/ml, Sigma-Aldrich, USA) and TroponinT (Rabbit, 1 mg/ml, Abcam, UK) at 1:400 and 1:200 dilution factor in PBS, respectively. Then, the samples were treated with Alexa Fluor 488 (Donkey anti-mouse, 2 mg/ml, Thermofisher, USA) and Alexa Fluor 555 (Donkey anti-rabbit, 2 mg/ml, Thermofisher, USA) at 1:400 dilution factor for the Alexa Fluor to PBS dilution in blocking buffer for 1 h at room temperature. After washing the samples with wash buffer (1 vol% donkey serum, 0.1 vol% Tween 20/1X in PBS), the nuclei were stained with NucBlue™ Fixed Cell ReadyProbes™ Reagent DAPI (Thermofisher, USA, original concentration of 5 µM in water) using 2 drops per ml in wash buffer for 5 min at room temperature. The stained samples were then imaged with a confocal microscope (Olympus FluoView FV1000, Japan).

### Real-time polymerase chain reaction

The expression of relevant genes was assessed using real-time polymerase chain reaction (RT-PCR) for cardiomyocytes and fibroblasts. The RNA was collected using a pure RNA kit (Thermo Fisher Scientific, USA). The purity and concentration of RNA were measured using a NanoDrop (Thermo Fisher Scientific, USA). RNA was converted into cDNA using the reverse cDNA Kit (Thermo Fisher Scientific, USA). Cardiac gene primers (ACTC1, MYH6, TNNT2, and GJa1) and fibroblast primers (Vtn, Tln1, and ACTA2) were purchased from Eurofins, USA. The SYBR Green master mix (Thermo Fisher Scientific, USA) was used for qRT-PCR reactions (Step one™ real-time system, applied biosystem, USA). The C_t_ method was used, using ACTB (Erofin, USA) as the housekeeping gene to quantify and compare the expression of genes.

## Data Availability

All data will be available by contacting the corresponding author.
